# Supplementary material for: Intracranial-Pressure-Monitoring-Assisted Management Associated with Favorable Outcomes in Moderate Traumatic Brain Injury Patients with a GCS of 9–11
Source: J Clin Med. 2022 Nov 10;11(22):6661. doi: 10.3390/jcm11226661 (PMC9694446; doi:10.3390/jcm11226661)
Supplement: Supplementary file 1 [file jcm-11-06661-s001.zip › Supplementary Table S1.pdf]

Supplementary Table S1. Patients' characteristics of non-ICP and ICP monitored

Characteristics	*Category*	*All patients (n=350)*	*Non-ICP monitored (n=205)*	*ICP monitored (n=145)*	*Z/T/χ^2*	*P-value*
At admission						
Age	IQ range	54 [42, 63]	54 [43, 63]	54[43, 63]	-0.273	0.786*
Sex	Female	95 (27.1%)	65 (68.4%)	30 (31.6%)	5.341	0.021
	Male	255(72.9%)	140 (54.7%)	115 (45.3%)		
COPD	No	314 (90.0%)	185 (58.9%)	129 (41.1%)	0.278	0.598
	Yes	36(10.0%)	20 (54.8%)	16 (45.2%)		
Hypertension	No	270 (77.4%)	165 (61.1%)	105 (38.9%)	3.471	0.062
	Yes	80 (22.6%)	40 (50.0%)	40 (50.0%)		
Coronary heart disease	No	334 (95.2%)	195 (58.4%)	139 (41.6%)	0.015	0.901
	Yes	16 (4.8%)	10 (60.5%)	6(39.5%)		
Diabetes	No	327 (93.5%)	190 (58.1%)	137 (41.9%)	0.260	0.610
	Yes	23 (6.5%)	15 (65.6%)	8 (34.4%)		
Aspirin	No	331 (94.8%)	195 (58.9%)	136(41.1%)	0.558	0.455
	Yes	19(5.2%)	10 (52.6%)	9 (47.4%)		
Clopidogrel	No	347 (99.4%)	203 (58.5%)	144 (41.5%)	0.000	1.000
	Yes	3 (0.6%)	2 (66.7%)	1 (33.3%)		
Anticoagulant	No	344(98.5%)	202(72.7%)	142 (41.3%)	0.149	0.699
	Yes	6 (1.5%)	3 (50.0%)	3 (50.0%)		
Alcohol abuse	No	319 (91.2%)	192 (60.2%)	127 (39.8%)	4.602	0.032
	Yes	31 (8.8%)	13 (42.2%)	18 (57.8%)		
Smoking history	No	296 (84.8%)	176 (59.5%)	120 (40.5%)	0.813	0.367
	Yes	54 (15.2%)	29 (53.8%)	25 (46.2%)		
GCS score	IQ range	10 [9, 11]	10 [10, 11]	10 [9, 10]	-3.593	0.001*
	>10	253 (72.3%)	166 (65.6%)	87 (34.4%)	18.651	<0.001
	≤10	97 (27.7%)	39 (40.2%)	58 (59.8%)		
ISS	IQ range	11 [11, 17]	11 [11, 14]	14 [11, 19]	-3.718	0.001*
	≥15	89 (25.4%)	38 (42.7%)	51 (57.3%)	12.395	<0.001
	<15	261 (74.6%)	167 (64.0%)	94 (36.0%)		
Injury mechanism	Motor vehicle	186 (53.3%)	102 (54.8%)	84 (45.2%)	4.799	0.181

	Pedestrian accident	106 (30.3%)	71(67.0%)	35 (33.0%)		
	Fall	47 (13.4%)	25 (53.2%)	22 (46.8%)		
	Assault	11 (3.1%)	7 (63.6%)	4(36.4%)		
Marshall's scale	Type I DI	13 (3.7%)	7 (53.8%)	6 (46.2%)	11.653	0.018△
	Type II DI	245 (70.2%)	151 (61.6%)	94 (38.4%)		
	Type III DI	19 (5.4%)	7 (36.8%)	12 (63.2%)		
	Type IV DI	10 (2.9%)	2 (20.0%)	8 (80.0%)		
	NEML	63 (17.8%)	38 (59.7%)	25 (40.3%)		
Midline shift (mm)	IQ range	0 [0, 1.9]	0 [0, 0]	0 [0, 3.45]	-4.471	0.001*
	≥2.5	86 (24.6%)	26 (30.2%)	60 (69.8%)	37.735	<0.001
	<2.5	264 (75.4%)	179 (67.8%)	85 (32.2%)		
IVH	No	311 (89.1%)	182 (58.5%)	129 (41.5%)	0.005	0.941
	Yes	39 (10.9%)	23 (59.9%)	16 (40.1%)		
tSAH modified Fisher scale	Grade 0	92 (26.4%)	57 (62.0%)	35 (38.0%)	12.915	0.005
	Grade 1	140 (40.1%)	93 (66.4%)	47 (33.6%)		
	Grade 2	87 (24.9%)	43 (49.4%)	44 (50.6%)		
	Grade 3	31 (8.6%)	12 (36.7%)	19 (63.3%)		
Skull fracture	No	125 (35.8%)	82(65.6%)	43 (34.4%)	4.097	0.043
	Yes	225 (64.2%)	123 (54.5%)	102 (45.5%)		
EDH	No	269 (77.1%)	160 (59.5%)	109 (40.5%)	0.509	0.475
	Yes	81(22.9%)	45 (56.5%)	36 (43.5%)		
SDH	No	187 (53.6%)	127(67.9%)	60 (32.1%)	14.851	<0.001
	Yes	163 (46.4%)	78 (46.5%)	85 (53.5%)		
Location of contusion (LOC)	None	79 (22.6%)	59 (74.7%)	20 (25.3%)	12.106	0.019
	Frontal	73 (20.9%)	44 (60.3%)	29 (39.7%)		
	Temporal	54 (15.5%)	30 (55.6%)	24 (44.4%)		
	Frontal and temporal	127 (36.4%)	70 (55.1%)	57 (44.9%)		

	Others` location	17 (4.6%)	9 (52.9.0%)	8 (47.1.0%)		
DAI	No	326 (93.4%)	191 (58.6.5%)	135 (41.4%)	0.038	0.846
	Yes	24 (6.6%)	14 (59.5%)	10 (40.5%)		
In ICU						
ICP monitoring time (h)	-	71.0±3.4	-	71.0±3.4	-	-
Intracranial infection	No	341 (97.4%)	201 (58.9%)	140 (41.1%)	0.280	0.597[b]
	Yes	9 (2.6%)	4 (44.4%)	5 (55.6%)		
ICP related hemorrhage	No	346 (98.9%)	205 (59.2%)	141 (40.8%)	-	0.008△
	Yes	4 (1.1%)	0 (0%)	4 (100%)		
ICU-LOS	IQ range	5 [3, 10]	6 [3, 10]	4 [2, 9]	-3.491	0.002*
LOS in hospital	IQ range	11 [4, 13]	10 [6, 12]	8 [5, 13]	-4.071	0.001*

DAI, Diffuse axonal injury, COPD, chronic obstructive pulmonary disease, ND, Neurological deterioration, LOS, length of stay

*P-value obtained by a nonparametric test

△P-value obtained by Fisher's exact test

Others` locations: Parietal/occipital/cerebellum
