# Supplementary material for: Intracranial-Pressure-Monitoring-Assisted Management Associated with Favorable Outcomes in Moderate Traumatic Brain Injury Patients with a GCS of 9–11
Source: J Clin Med. 2022 Nov 10;11(22):6661. doi: 10.3390/jcm11226661 (PMC9694446; doi:10.3390/jcm11226661)
Supplement: Supplementary file 1 [file jcm-11-06661-s001.zip › Supplementary Table S10.pdf]

**Supplementary Table S10.** The impact of neurological deterioration on GOSE≤4.

| *Characteristics* | *Category* | *All patients (n=350)* | *GOSE>4 (n=229)* | *GOSE≤4 (n=121)* | $\chi^2$ | *P-value* |
|---|---|---|---|---|---|---|
| ND | No | 219 (62.8%) | 181 (82.6%) | 38 (17.4%) | 77.634 | <0.001 |
| | Yes | 131 (37.2%) | 48 (36.2%) | 83 (63.8%) | | |
