# Supplementary material for: Intracranial-Pressure-Monitoring-Assisted Management Associated with Favorable Outcomes in Moderate Traumatic Brain Injury Patients with a GCS of 9–11
Source: J Clin Med. 2022 Nov 10;11(22):6661. doi: 10.3390/jcm11226661 (PMC9694446; doi:10.3390/jcm11226661)
Supplement: Supplementary file 1 [file jcm-11-06661-s001.zip › Supplementary Table S11.pdf]

Supplementary Table S11. The influence of ICP-monitored therapy on potential neurological deterioration reasons.

Characteristics	*Category*	*All patients (n=350)*	*Non-ICP monitored (n=205)*	*ICP monitored (n=145)*	χ^2	*P-value*
Potential ND causes	No potential ND reasons.	210 (59.7%)	110 (52.6%)	100 (47.4%)	18.787	< 0.001
	Hematoma expansion	46 (13.4%)	22 (46.8%)	24 (53.2%)		
	Cerebral edema aggravation	83 (23.7%)	64 (77.1%)	19 (22.9%)		
	General deterioration reasons	11 (3.2%)	6 (54.6%)	5 (45.4%)		
