# Supplementary material for: Intracranial-Pressure-Monitoring-Assisted Management Associated with Favorable Outcomes in Moderate Traumatic Brain Injury Patients with a GCS of 9–11
Source: J Clin Med. 2022 Nov 10;11(22):6661. doi: 10.3390/jcm11226661 (PMC9694446; doi:10.3390/jcm11226661)
Supplement: Supplementary file 1 [file jcm-11-06661-s001.zip › Supplementary Table S12.pdf]

Supplementary Table S12. The influence of ICP-monitored therapy on neurological deterioration reasons.

Characteristics	*Category*	*All patients (n=131)*	*Non-ICP monitored (n=92)*	*ICP monitored (n=39)*	χ^2	*P-value*
ND causes	Hematoma expansion	42 (32.3%)	22 (52.4%)	20 (47.6%)	9.185	0.010
	Cerebral edema aggravation	78 (59.5%)	61 (78.2%)	17 (21.8%)		
	General deterioration	11 (8.2%)	6 (54.6%)	5 (45.4%)		
