# Supplementary material for: Intracranial-Pressure-Monitoring-Assisted Management Associated with Favorable Outcomes in Moderate Traumatic Brain Injury Patients with a GCS of 9–11
Source: J Clin Med. 2022 Nov 10;11(22):6661. doi: 10.3390/jcm11226661 (PMC9694446; doi:10.3390/jcm11226661)
Supplement: Supplementary file 1 [file jcm-11-06661-s001.zip › Supplementary Table S13.pdf]

Supplementary Table S13. The 72h ICP management intensity between ICP monitored and control group.

Characteristic	*Non-ICP monitored (n=205)*	*ICP monitored (n=145)*	Z	*P-value*
72h-TILscore (total)	9 (8, 11)	13 (9, 17)	-8.388	<0.001
