# Supplementary material for: Intracranial-Pressure-Monitoring-Assisted Management Associated with Favorable Outcomes in Moderate Traumatic Brain Injury Patients with a GCS of 9–11
Source: J Clin Med. 2022 Nov 10;11(22):6661. doi: 10.3390/jcm11226661 (PMC9694446; doi:10.3390/jcm11226661)
Supplement: Supplementary file 1 [file jcm-11-06661-s001.zip › Supplementary Table S14.pdf]

Supplementary Table S14. The impact of 72h ICP management intensity on GOSE.

Characteristic	*GOSE>4 (n=229)*	*GOSE≤4 (n=121)*	Z	*P-value*
72h-TILscore (total)	12 (9, 16)	10 (9, 13)	-3.000	0.003
