# Supplementary material for: Intracranial-Pressure-Monitoring-Assisted Management Associated with Favorable Outcomes in Moderate Traumatic Brain Injury Patients with a GCS of 9–11
Source: J Clin Med. 2022 Nov 10;11(22):6661. doi: 10.3390/jcm11226661 (PMC9694446; doi:10.3390/jcm11226661)
Supplement: Supplementary file 1 [file jcm-11-06661-s001.zip › Supplementary Table S16.pdf]

Supplementary Table S16. The AUC of different ICP characteristics on GOSE≤4.

	AUC	95%CI	P	Cutoff	Sensitivity	Specificity
Mean ICP (mmHg）	0.698	0.606~0.789	<0.001	>15.8	51.01	86.21
Number of ICP>15mmHg	0.681	0.587~0.774	<0.001	>25.5	58.80	75.54
Number of ICP>20 mmHg	0.660	0.561~0.759	0.002	>5.5	43.31	95.37
