# Supplementary material for: Intracranial-Pressure-Monitoring-Assisted Management Associated with Favorable Outcomes in Moderate Traumatic Brain Injury Patients with a GCS of 9–11
Source: J Clin Med. 2022 Nov 10;11(22):6661. doi: 10.3390/jcm11226661 (PMC9694446; doi:10.3390/jcm11226661)
Supplement: Supplementary file 1 [file jcm-11-06661-s001.zip › Supplementary Table S2.pdf]

Supplementary Table S2. The AUC of continuous variables was investigated by using univariate analysis with ICP and non-ICP groups.

	AUC	95%CI	P	Cutoff	Sen (%)	Spe (%)
ISS score	0.591	0.529~0.653	0.004	≥ 15	35.80	81.50
GCS score	0.647	0.572~0.701	0.001	≤ 10	77.20	43.70
Midline shift (mm)	0.654	0.594~0.714	< 0.001	≥ 2.5	46.9	83.4
