# Supplementary material for: Intracranial-Pressure-Monitoring-Assisted Management Associated with Favorable Outcomes in Moderate Traumatic Brain Injury Patients with a GCS of 9–11
Source: J Clin Med. 2022 Nov 10;11(22):6661. doi: 10.3390/jcm11226661 (PMC9694446; doi:10.3390/jcm11226661)
Supplement: Supplementary file 1 [file jcm-11-06661-s001.zip › Supplementary Table S3.pdf]

Supplementary Table S3. The results of the multivariate logistic regression analysis of ICP monitored.

Characteristics		OR	95%CI	P-value
GCS score	≤10	1.751	1.216-3.020	0.003
	>10*	1		
ISS	≥15	1.535	0.853-2.762	0.153
	<15*	1		
Midline shift (mm)	≥2.5	3.916	2.076-7.386	<0.001
	<2.5*	1		
Sex	Male	1.177	0.666-2.08	0.575
	Female*	1		
Alcohol abuse	Yes	1.822	0.772-4.304	0.171
	No*	1		
Marshall's scale	Type II DI	2.123	0.517-8.713	0.296
	Type III DI	0.96	0.502-1.833	0.901
	Type IV DI	1.007	0.268-3.786	0.991
	NEML	1.221	0.203-7.356	0.828
	Type I DI*	1		
SDH	Yes	1.772	1.065-2.949	0.028
	No*	1		
tSAH modified Fisher scale	Grade 1	0.884	0.484-1.27	0.14
	Grade 2	1.328	0.731-2.823	0.18
	Grade 3	1.441	0.866-3.171	0.1
	Grade 0*	1		
Skull fracture	Yes	1.476	0.843-2.552	0.175
	No*	1		
Location of contusion (LOC)	Frontal	0.642	0.181-2.274	0.492
	Temporal	0.671	0.196-2.293	0.524
	Frontal and temporal	0.739	0.206-2.648	0.642
	Others' location	0.915	0.28-2.993	0.883
	None*			

OR odds ratio; *95% CI* 95%, confidence interval; * control group
