# Supplementary material for: Intracranial-Pressure-Monitoring-Assisted Management Associated with Favorable Outcomes in Moderate Traumatic Brain Injury Patients with a GCS of 9–11
Source: J Clin Med. 2022 Nov 10;11(22):6661. doi: 10.3390/jcm11226661 (PMC9694446; doi:10.3390/jcm11226661)
Supplement: Supplementary file 1 [file jcm-11-06661-s001.zip › Supplementary Table S4.pdf]

**Supplementary Table S4.** The 6-Month outcome of all patients.

| *Characteristics* | *Category* | *All patients (n=350)* | *Non-ICP monitored (n=205)* | *ICP monitored (n=145)* | $\chi^2$ | *P-value* |
|---|---|---|---|---|---|---|
| 6-Month outcome | Dead (GOSE-1) | 54 (15.4%) | 40(19.5%) | 14 (9.7%) | 6.419 | 0.011 |
| | Unfavorable survival (GOSE-1~4 | 121 (34.6%) | 84 (41.0%) | 37 (25.5%) | 9.176 | 0.002 |
| | Favorable survival (GOSE-5~8) | 229 (65.4%) | 121 (59.0%) | 108 (74.5%) | | |
