# Supplementary material for: Intracranial-Pressure-Monitoring-Assisted Management Associated with Favorable Outcomes in Moderate Traumatic Brain Injury Patients with a GCS of 9–11
Source: J Clin Med. 2022 Nov 10;11(22):6661. doi: 10.3390/jcm11226661 (PMC9694446; doi:10.3390/jcm11226661)
Supplement: Supplementary file 1 [file jcm-11-06661-s001.zip › Supplementary Table S5.pdf]

Supplementary Table S5. Univariate analysis results of GOSE≤4.

Characteristics	*Category*	*All patients (n=350)*	*GOSE>4 (n=229)*	*GOSE≤4 (n=121)*	*Z/T/χ²*	*P-value*
Age (year)	IQ range	54 [42, 63]	52 [42, 62]	56[44, 64]	-1.438	0.150*
Sex	Female	95 (27.1%)	64 (67.4%)	31 (32.6%)	0.178	0.673
	Male	255(72.9%)	165 (63.7%)	90 (36.3%)		
COPD	No	314 (90.0%)	202 (64.3%)	112 (35.7%)	2.293	0.130
	Yes	36(10.0%)	27 (74.8%)	9 (25.2%)		
Hypertension	No	270 (77.4%)	177 (65.6%)	93 (34.4%)	0.002	0.965
	Yes	80 (22.6%)	52 (65.8.0%)	28 (34.2%)		
Coronary heart disease	No	334 (95.2%)	221 (66.2%)	113 (33.8%)	1.048	0.306
	Yes	16 (4.8%)	8 (50.0%)	8(50.0%)		
Diabetes	No	327 (93.5%)	215 (65.7%)	112 (34.3%)	0.041	0.840
	Yes	23 (6.5%)	14 (63.6%)	9 (36.4%)		
Aspirin	No	331 (94.8%)	219 (66.2%)	112(33.8%)	0.851	0.356
	Yes	19(5.2%)	10 (52.6%)	9 (47.4%)		
Clopidogrel	No	347 (99.4%)	228 (65.7%)	119 (34.3%)	1.444	0.229[b]
	Yes	3 (0.6%)	1 (33.3%)	2 (66.7%)		
Anticoagulant	No	344(98.5%)	226(65.7%)	118 (34.3%)	0.071	0.790[b]
	Yes	6 (1.5%)	3 (50.0%)	3 (50.0%)		
Alcohol abuse	No	319 (91.2%)	207 (64.9%)	112 (35.1%)	0.866	0.352
	Yes	31 (8.8%)	22 (73.3%)	9 (26.7%)		
Smoking history	No	296 (84.8%)	188 (63.5%)	108 (36.5%)	3.619	0.061
	Yes	54 (15.2%)	41 (75.4%)	13 (24.6%)		
GCS score	IQ range	10 [9, 11]	10 [10, 11]	10 [9, 10]	-3.914	<0.001*
ISS	IQ range	11 [11, 17]	11 [11, 16]	14 [11, 17]	-0.662	0.508*
Injury mechanism	Motor vehicle	186 (53.3%)	124 (66.7%)	62 (33.3%)	1.850	0.604
	Pedestrian accident	106 (30.3%)	69(65.1%)	37 (34.9%)		
	Fall	47 (13.4%)	28 (59.6%)	19 (40.4%)		
	Assault	11 (3.1%)	8 (68.6%)	3(31.4%)		
Marshall's scale	Type I DI	13 (3.7%)	12 (92.3%)	1 (3.7%)	26.427	<0.001△
	Type II DI	245 (70.2%)	174 (71.0%)	71 (29.0%)		
	Type III	19 (5.4%)	8 (42.1%)	11 (57.9%)		

	DI					
	Type IV DI	10 (2.9%)	2 (20.0%)	8 (80.0%)		
	NEML	63 (17.8%)	33 (50.6%)	30 (49.4%)		
Midline shift (mm)	IQ range	0 [0, 1.4]	0 [0, 0]	0 [0, 2.6]	-3.542	<0.001*
IVH	No	311 (89.1%)	207 (69.5%)	104 (30.5%)	1.908	0.167
	Yes	39 (10.9%)	22 (55.3%)	17 (44.7%)		
tSAH modified Fisher scale	Grade 0	92 (26.4%)	64 (69.6%)	28 (30.4%)	4.146	0.246
	Grade 1	140 (40.1%)	90 (64.3%)	50 (35.7%)		
	Grade 2	87 (24.9%)	59 (67.8%)	28 (32.2%)		
	Grade 3	31 (8.6%)	17 (56.7%)	14 (43.3%)		
Skull fracture	No	125 (35.8%)	83(66.4%)	42 (33.6%)	0.099	0.754
	Yes	225 (64.2%)	146 (64.7%)	79 (35.3%)		
EDH	No	269 (77.1%)	180 (66.9%)	89 (33.1%)	1.302	0.254
	Yes	81(22.9%)	49 (60.7%)	32 (39.3%)		
SDH	No	187 (53.6%)	136(72.7%)	51 (27.3%)	9.734	0.002
	Yes	163 (46.4%)	93 (56.8%)	70 (43.2%)		
Location of contusion (LOC)	None	79 (22.6%)	58 (73.4%)	21 (26.6%)	11.330	0.023
	Frontal	73 (20.9%)	54 (73.3%)	19 (26.7%)		
	Temporal	54 (15.5%)	34 (62.6%)	20 (37.4%)		
	Frontal and temporal	127 (36.4%)	70 (55.1%)	57 (44.9%)		
	Others' location	17 (4.6%)	13 (72.5%)	4 (27.5%)		
DAI	No	326 (93.4%)	212 (65.0%)	114 (35.0%)	0.195	0.659
	Yes	24 (6.6%)	17 (70.8%)	7 (29.2%)		
ICP monitored	No	205 (58.5%)	121 (58.8%)	84 (41.2%)	9.176	0.002
	Yes	145 (41.5%)	108 (74.5%)	37 (25.5%)		

DAI, Diffuse axonal injury, COPD, chronic obstructive pulmonary disease, ND, Neurological deterioration

*P-value obtained by a nonparametric test

[b]P-value obtained by continuity correction Chi-square test

[△]P-value obtained by Fisher’s exact test

Others' locations: Parietal/occipital/cerebellum
