# Supplementary material for: Intracranial-Pressure-Monitoring-Assisted Management Associated with Favorable Outcomes in Moderate Traumatic Brain Injury Patients with a GCS of 9–11
Source: J Clin Med. 2022 Nov 10;11(22):6661. doi: 10.3390/jcm11226661 (PMC9694446; doi:10.3390/jcm11226661)
Supplement: Supplementary file 1 [file jcm-11-06661-s001.zip › Supplementary Table S6.pdf]

Supplementary Table S6. Univariate analysis results of GOSE≤4 with GCS 11 patients.

Characteristics	*Category*	*All patients (n=253)*	*GOSE>4 (n=174)*	*GOSE≤4 (n=79)*	$Z/T/\chi^2$	*P-value*
Age (year)	IQ range	53 [42, 62]	53 [43, 64]	55[31, 62]	-1.657	0.098*
Sex	Female	79 (31.2%)	56 (70.9%)	23 (29.1%)	0.238	0.625
	Male	174(68.8%)	118 (67.8%)	56 (32.2%)		
COPD	No	229 (90.5%)	156 (68.1%)	73 (31.9%)	0.478	0.489
	Yes	24(9.5%)	18 (75.0%)	6 (25.0%)		
Hypertension	No	199 (78.7%)	137 (68.8%)	62 (31.2%)	0.002	0.963
	Yes	54 (21.3%)	37 (68.5%)	17 (31.5%)		
Coronary heart disease	No	242 (95.7%)	170 (70.2%)	72 (29.8%)	4.158	0.041[b]
	Yes	11 (4.3%)	4 (36.4%)	7(63.6%)		
Diabetes	No	240 (94.9%)	165 (68.8%)	75 (31.3%)	0.001	0.971[b]
	Yes	13 (5.1%)	9 (69.2%)	4 (30.8%)		
Aspirin	No	242 (95.7%)	168 (69.4%)	74(30.6%)	1.084	0.298
	Yes	11(4.3%)	6 (54.5%)	5 (45.5%)		
Clopidogrel	No	251 (99.2%)	174 (69.3%)	77 (30.7%)	-	0.097△
	Yes	2 (0.8%)	0 (0%)	2 (100%)		
Anticoagulant	No	249(98.4%)	171(68.7%)	78 (31.3%)	0.001	0.971[b]
	Yes	4 (1.6%)	3 (75.0%)	1 (25.0%)		
Alcohol abuse	No	230 (90.9%)	155 (67.4%)	75 (32.6%)	1.602	0.206[b]
	Yes	23 (9.1%)	19 (82.6%)	4 (17.4%)		
Smoking history	No	211 (83.4%)	144 (68.2%)	67 (31.8%)	0.165	0.684
	Yes	42 (16.6%)	30 (71.4%)	12 (28.6%)		
ISS	IQ range	11 [11, 14]	11 [11, 14]	11 [11, 14]	-0.825	0.409*
Injury mechanism	Motor vehicle	142 (56.1%)	102 (71.8%)	40 (28.2%)	1.804	0.614
	Pedestrian accident	72 (28.5%)	48(66.7%)	24 (33.3%)		
	Fall	33 (13.0%)	20 (60.6%)	13 (39.4%)		
	Assault	6 (2.4%)	4 (66.7%)	2(33.3%)		
Marshall's scale	Type I DI	7 (2.8%)	7 (100%)	0 (0%)	11.267	0.017△
	Type II DI	183 (72.3%)	133 (72.7%)	50 (27.3%)		
	Type III DI	14 (5.5%)	8 (57.1%)	6 (42.9%)		

	Type IV DI	5 (2.0%)	2 (40.0%)	3 (60.0%)		
	NEML	44 (17.4%)	24 (54.5%)	20 (45.5%)		
Midline shift (mm)	IQ range	0 [0, 2.15]	0 [0, 2.13]	0 [0, 2.2]	-0.392	0.695*
IVH	No	228 (90.1%)	159 (69.7%)	69 (30.3%)	0.995	0.319
	Yes	25 (9.9%)	15 (60.0%)	10 (40.0%)		
tSAH modified Fisher scale	Grade 0	71 (28.1%)	48 (67.6%)	23 (32.4%)	7.853	0.046
	Grade 1	99 (39.1%)	66 (66.7%)	33 (33.3%)		
	Grade 2	64 (25.3%)	51 (79.7%)	13 (20.3%)		
	Grade 3	19 (7.5%)	9 (47.4%)	10 (52.6%)		
Skull fracture	No	94 (37.2%)	64(68.1%)	30 (31.9%)	0.039	0.889
	Yes	159 (62.8%)	110 (69.2%)	49 (30.8%)		
EDH	No	194 (76.7%)	135 (69.6%)	59 (30.4%)	0.256	0.613
	Yes	59 (23.3%)	39 (66.1%)	20 (33.9%)		
SDH	No	147 (58.1%)	112(76.2%)	35 (23.8%)	8.985	0.003
	Yes	106 (41.9%)	62 (58.5%)	44 (41.5%)		
Location of contusion (LOC)	None	51 (20.2%)	46 (90.2%)	5 (9.8%)	17.601	0.001
	Frontal	53 (20.9%)	39 (73.6%)	14 (26.4%)		
	Temporal	27 (10.7%)	19 (70.4%)	8 (29.6%)		
	Frontal and temporal	112 (44.3%)	64 (57.1%)	48 (42.9%)		
	Others' location	10 (4.0%)	6 (60.0%)	4 (40.0%)		
DAI	No	236 (93.3%)	161 (68.2%)	75 (31.8%)	0.508	0.474[b]
	Yes	17 (6.7%)	13 (76.5%)	4 (23.5%)		
ICP monitored	No	166 (65.6%)	114 (68.7%)	52 (31.3%)	0.306	0.816
	Yes	87 (34.4%)	60 (69.0%)	27 (31.0%)		

DAI, Diffuse axonal injury, COPD, chronic obstructive pulmonary disease, ND, Neurological deterioration

*P-value obtained by a nonparametric test

[b]P-value obtained by continuity correction Chi-square test

△P-value obtained by Fisher's exact test

Others' locations: Parietal/occipital/cerebellum
