# Supplementary material for: Intracranial-Pressure-Monitoring-Assisted Management Associated with Favorable Outcomes in Moderate Traumatic Brain Injury Patients with a GCS of 9–11
Source: J Clin Med. 2022 Nov 10;11(22):6661. doi: 10.3390/jcm11226661 (PMC9694446; doi:10.3390/jcm11226661)
Supplement: Supplementary file 1 [file jcm-11-06661-s001.zip › Supplementary Table S7.pdf]

Supplementary Table S7. Univariate analysis results of GOSE≤4 with GCS 9-10 patients.

Characteristics	*Category*	*All patients (n=97)*	*GOSE>4 (n=55)*	*GOSE≤4 (n=42)*	*Z/T/χ²*	*P-value*
Age (year)	IQ range	55 [42, 64]	52 [41, 60]	56[44, 64]	-0.361	0.718*
Sex	Female	16 (16.5%)	9 (56.3%)	7 (43.8%)	0.002	0.968
	Male	81(83.5%)	46 (56.8%)	35 (43.2%)		
COPD	No	86 (88.7%)	48 (55.8%)	38 (44.2%)	0.029	0.865[b]
	Yes	11(11.3%)	7 (63.6%)	4 (36.4%)		
Hypertension	No	72 (74.2%)	39 (54.2%)	33 (45.8%)	0.731	0.393
	Yes	25 (25.8%)	16 (64.0%)	9 (36.0%)		
Coronary heart disease	No	93 (95.9%)	52 (55.9%)	41 (44.1%)	0.057	0.811[b]
	Yes	4 (4.1%)	3 (75.0%)	1(25.0%)		
Diabetes	No	87 (89.7%)	48 (55.2%)	39 (44.8%)	0.313	0.576[b]
	Yes	10 (10.3%)	7 (70.0%)	3 (30.0%)		
Aspirin	No	90 (92.8%)	52 (57.8%)	38(42.2%)	0.138	0.710[b]
	Yes	7(7.2%)	3 (42.9%)	4 (57.1%)		
Clopidogrel	No	97 (100%)	55 (56.7%)	42 (43.3%)	-	-
	Yes	0 (0%)	0 (0%)	0 (0%)		
Anticoagulant	No	96(99.0%)	55(56.3%)	41 (43.8%)	-	0.990△
	Yes	1 (1.0%)	0 (0%)	1 (100.0%)		
Alcohol abuse	No	90 (92.8%)	50 (55.6%)	40 (44.4%)	0.177	0.674[b]
	Yes	7 (7.2%)	5 (71.4%)	2 (28.6%)		
Smoking history	No	86 (88.7%)	49 (57.0%)	37 (43.0%)	0.023	0.878
	Yes	11 (11.3%)	6 (54.5%)	5 (45.5%)		
ISS	IQ range	14 [11, 17]	14 [11, 17]	14 [11, 19]	-0.462	0.708*
Injury mechanism	Motor vehicle	45 (46.4%)	24 (53.3%)	21 (46.7%)	1.091	0.779
	Pedestrian accident	34 (35.1%)	19(55.9%)	15 (44.1%)		
	Fall	14 (14.4%)	9 (64.3%)	5 (35.7%)		
	Assault	4 (4.1%)	3 (75.0%)	1(25.0%)		
Marshall's scale	Type I DI	6 (6.2%)	5 (83.3%)	1 (16.7%)	14.896	0.002△
	Type II DI	62 (63.9%)	41 (66.1%)	21 (33.9%)		
	Type III DI	6 (6.2%)	1 (16.7%)	5 (83.3%)		
	Type IV DI	5 (5.2%)	0 (0%)	5 (100.0%)		
	NEML	18 (18.6%)	8 (44.4%)	10 (55.6%)		
Midline shift (mm)	IQ range	0 [0, 2.95]	0 [0, 2.40]	1.9 [0, 3.25]	-2.590	0.010*
IVH	No	84 (86.6%)	49 (58.3%)	35 (41.7%)	0.680	0.410
	Yes	13 (13.4%)	6 (46.2%)	7 (53.8%)		
tSAH modified	Grade 0	21 (21.6%)	16 (76.2%)	5 (23.8%)	7.946	0.043

Fisher scale						
	Grade 1	42 (43.3%)	25 (59.5%)	17 (40.5%)		
	Grade 2	23 (23.7%)	8 (34.8%)	15 (65.2%)		
	Grade 3	11 (11.3%)	6 (54.5%)	5 (45.5%)		
Skull fracture	No	31 (32.0%)	19(61.3%)	12 (38.7%)	0.391	0.661
	Yes	66 (68.0%)	36 (54.5%)	30 (45.5%)		
EDH	No	76 (78.4%)	46 (60.5%)	30 (39.5%)	2.092	0.148
	Yes	21(21.6%)	9 (42.9%)	12 (57.1%)		
SDH	No	41 (42.3%)	31(75.6%)	10 (24.4%)	10.342	0.001
	Yes	56 (57.7%)	24 (42.9%)	32 (57.1%)		
Location of contusion (LOC)	None	14 (14.4%)	11 (84.6%)	2 (15.4%)	13.008	0.009[△]
	Frontal	16 (16.5%)	12 (75.0%)	4 (25.0%)		
	Temporal	15 (15.5%)	10 (66.7%)	5 (33.3%)		
	Frontal and temporal	46 (47.4%)	19 (40.4%)	28 (59.6%)		
	Others’ location	6 (6.2%)	3 (50.0%)	3(50.0%)		
DAI	No	91 (93.8%)	51 (56.0%)	40 (44.0%)	0.063	0.802[b]
	Yes	6 (6.2%)	4 (66.7%)	2 (33.3%)		
ICP monitored	No	39 (40.2%)	13 (33.3%)	26 (66.7%)	11.994	0.001
	Yes	58 (59.8%)	42 (72.4%)	16 (27.6%)		

DAI, Diffuse axonal injury, COPD, chronic obstructive pulmonary disease, ND, Neurological deterioration

*P-value obtained by a nonparametric test

[b]P-value obtained by continuity correction Chi-square test

[△]P-value obtained by Fisher’s exact test

Others’ locations: Parietal/occipital/cerebellum
