# Supplementary material for: Intracranial-Pressure-Monitoring-Assisted Management Associated with Favorable Outcomes in Moderate Traumatic Brain Injury Patients with a GCS of 9–11
Source: J Clin Med. 2022 Nov 10;11(22):6661. doi: 10.3390/jcm11226661 (PMC9694446; doi:10.3390/jcm11226661)
Supplement: Supplementary file 1 [file jcm-11-06661-s001.zip › Supplementary Table S8.pdf]

Supplementary Table S8. The results of the multivariate logistic regression analysis of GOSE≤4 with GCS9-10 patients.

Characteristics		OR	95%CI	P-value
ICP monitored	Yes	0.565	0.314-0.837	0.001
	No*	1		
Midline shift (mm)		1.229	0.992-1.923	0.052
Marshall's scale	Type II DI	2.393	0.323-28.829	0.154
	Type III DI	22.123	1.963-235.421	0.010
	Type IV DI	68.877	5.185-1767.872	0.003
	NEML	9.683	2.931-121.438	0.034
	Type I DI*	1		
SDH	Yes	3.115	1.743-4.599	0.004
	No*	1		
Location of contusion (LOC)	Frontal	1.589	0.847-13.253	0.059
	Temporal	1.934	1.133-9.467	0.041
	Frontal and temporal	3.214	1.921-4.654	0.001
	Others' location	1.389	0.702-14.345	0.249
	None*	1		
tSAH modified Fisher scale	Grade 1	1.561	0.821-8.145	0.143
	Grade 2	2.389	0.899-6.345	0.049
	Grade 3	1.956	0.902-10.041	0.109
	Grade 0*			

OR odds ratio; *95% CI* 95%, confidence interval; * control group
