# Supplementary material for: Intracranial-Pressure-Monitoring-Assisted Management Associated with Favorable Outcomes in Moderate Traumatic Brain Injury Patients with a GCS of 9–11
Source: J Clin Med. 2022 Nov 10;11(22):6661. doi: 10.3390/jcm11226661 (PMC9694446; doi:10.3390/jcm11226661)
Supplement: Supplementary file 1 [file jcm-11-06661-s001.zip › Supplementary Table S9.pdf]

Supplementary Table S9. The influence of ICP-monitored therapy on neurological deterioration.

Characteristics	*Category*	*All patients (n=350)*	*Non-ND (n=219)*	*ND (n=131)*	χ^2	*P-value*
ICP monitored	No	205 (58.6%)	113 (55.4%)	92 (44.6%)	11.375	0.010
	Yes	145 (41.4%)	106 (73.1%)	39 (26.9%)		
