# Supplementary figures and images for: Intracranial-Pressure-Monitoring-Assisted Management Associated with Favorable Outcomes in Moderate Traumatic Brain Injury Patients with a GCS of 9–11
Source: J Clin Med. 2022 Nov 10;11(22):6661. doi: 10.3390/jcm11226661 (PMC9694446; doi:10.3390/jcm11226661)

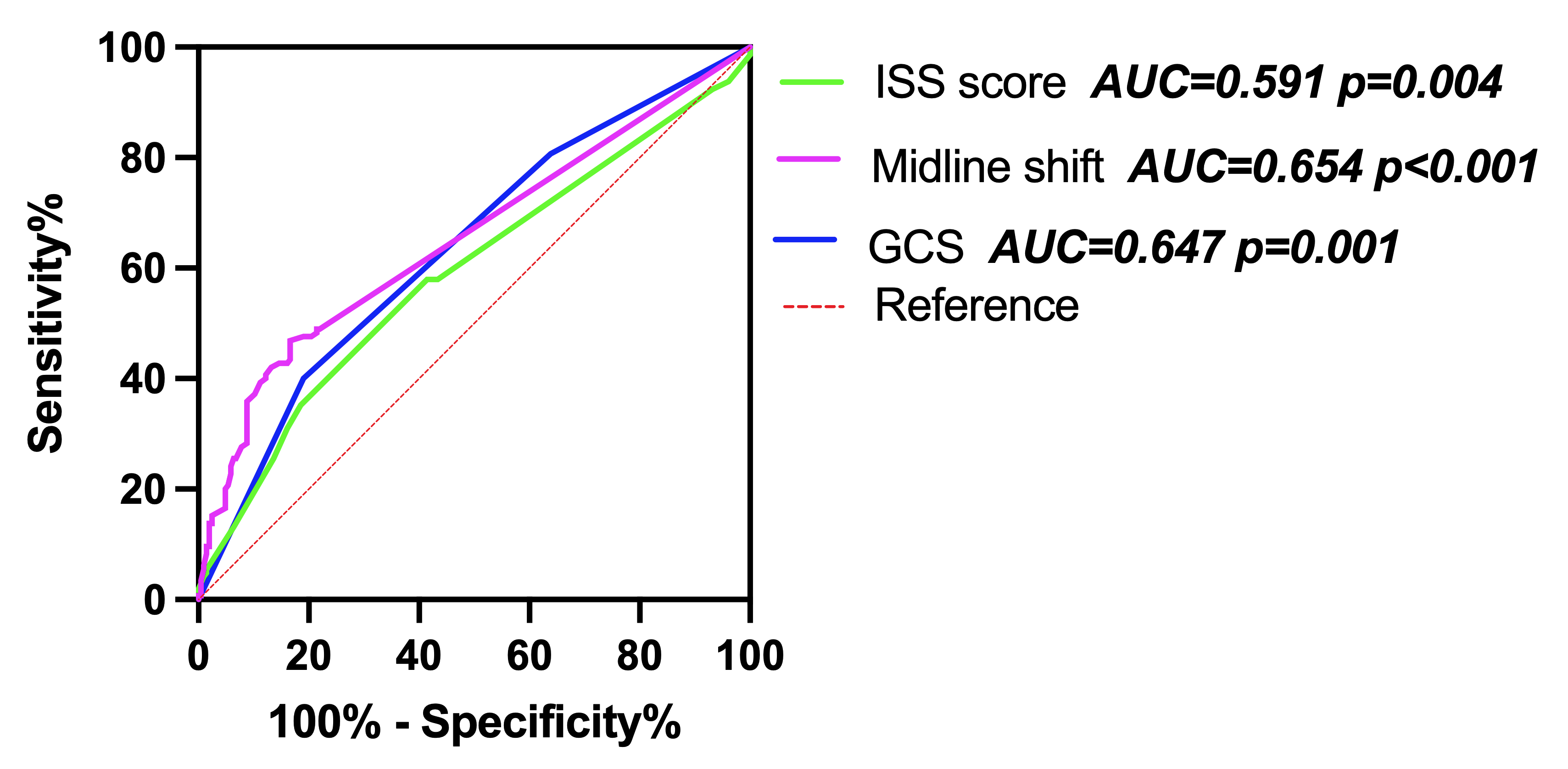

Supplement: Supplementary file 1 [file jcm-11-06661-s001.zip › Supplementary Figure S1.tiff]

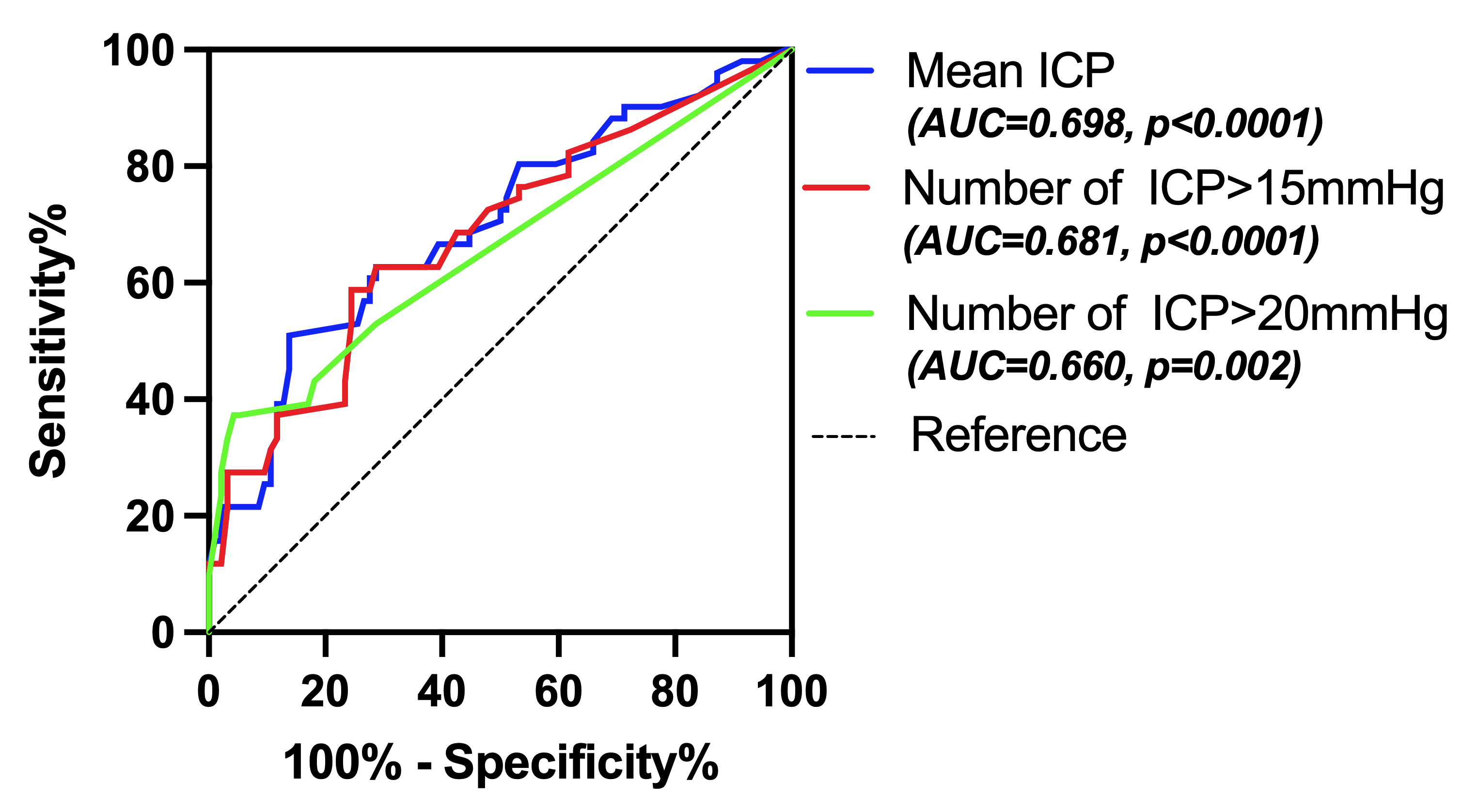

Supplement: Supplementary file 1 [file jcm-11-06661-s001.zip › Supplementary Figure S2.tiff]
